# Current and Future Applications of Arterial Spin Labeling MRI in Cerebral Arteriovenous Malformations

**DOI:** 10.3390/biomedicines12040753

**Published:** 2024-03-28

**Authors:** Matteo De Simone, Marco Maria Fontanella, Anis Choucha, Karl Schaller, Paolo Machi, Giuseppe Lanzino, Philippe Bijlenga, Felix T. Kurz, Karl-Olof Lövblad, Lucio De Maria

**Affiliations:** 1 Department of Medicine, Surgery and Dentistry “Scuola Medica Salernitana”, University of Salerno, Via S. Allende, 84081 Baronissi, Italy; 2 Division of Neurosurgery, Department of Medical and Surgical Specialties, Radiological Sciences and Public Health, University of Brescia, Piazza Spedali Civili 1, 25123 Brescia, Italy; marco.fontanella@unibs.it (M.M.F.); luciodemaria@gmail.com (L.D.M.); 3 Department of Neurosurgery, Aix Marseille University, APHM, UH Timone, 13005 Marseille, France; anis.c13@gmail.com; 4 Laboratory of Biomechanics and Application, UMRT24, Gustave Eiffel University, Aix Marseille University, 13005 Marseille, France; 5 Division of Neurosurgery, Diagnostic Department of Clinical Neurosciences, Geneva University Hospitals (HUG), Rue Gabrielle-Perret-Gentil 4, 1205 Geneva, Switzerland; karl.schaller@hcuge.ch (K.S.); philippe.bijlenga@hcuge.ch (P.B.); 6 Division of Interventional Neuroradiology, Department of Radiology and Medical Informatic, Geneva University Hospitals (HUG), Rue Gabrielle-Perret-Gentil 4, 1205 Geneva, Switzerland; paolo.machi@hcuge.ch (P.M.); felixtobias.kurz@hcuge.ch (F.T.K.); karlolof.lovblad@hug.ch (K.-O.L.); 7 Department of Neurosurgery and Interventional Neuroradiology, Mayo Clinic, 200 1st St SW, Rochester, MN 55905, USA; lanzino.giuseppe@mayo.edu

**Keywords:** ASL, AVM, DVA, arteriovenous shunting, NCE 4D MRA, 4D-S-PACK

## Abstract

Arterial spin labeling (ASL) has emerged as a promising noninvasive tool for the evaluation of both pediatric and adult arteriovenous malformations (AVMs). This paper reviews the advantages and challenges associated with the use of ASL in AVM assessment. An assessment of the diagnostic workup of AVMs and their variants in both adult and pediatric populations is proposed. Evaluation after treatments, whether endovascular or microsurgical, was similarly examined. ASL, with its endogenous tracer and favorable safety profile, offers functional assessment and arterial feeder identification. ASL has demonstrated strong performance in identifying feeder arteries and detecting arteriovenous shunting, although some studies report inferior performance compared with digital subtraction angiography (DSA) in delineating venous drainage. Challenges include uncertainties in sensitivity for specific AVM features. Detecting AVMs in challenging locations, such as the apical cranial convexity, is further complicated, demanding careful consideration due to the risk of underestimating total blood flow. Navigating these challenges, ASL provides a noninvasive avenue with undeniable merits, but a balanced approach considering its limitations is crucial. Larger-scale prospective studies are needed to comprehensively evaluate the diagnostic performance of ASL in AVM assessment.

## 1. Introduction

Arteriovenous malformations (AVMs) are a rare condition of abnormal shunt involving tangles of dysplastic arteries and veins that converge to form a vascular nidus without the interposition of a capillary bed [1]. They are rare cerebrovascular diseases that can cause intracranial hemorrhage. The incidence of AVMs ranges from 1.12 to 1.42 cases per 100,000 person years, with 38–68% of new cases coming to medical attention with a picture of cerebral hemorrhage. In addition, annual hemorrhage rates in untreated AVMs have been estimated at 2.10–4.12% [2]. Meta-analyses on the topic found that the increased risk of future rupture was associated with factors such as a previous rupture, location of the AVM in deep brain structures, and exclusive deep venous drainage [3,4].

The pathophysiology of AVMs involves a disturbance in the normal regulation of blood flow and pressure within the brain. The high-flow arterial blood entering the low-flow venous system can cause venous hypertension, leading to venous dilatation and an increased risk of hemorrhage. Additionally, the lack of a capillary bed within the AVM disrupts the normal oxygen and nutrient exchange between arteries and veins, further contributing to tissue hypoxia and ischemia [5]. Furthermore, AVMs can trigger a phenomenon known as “steal”, where the abnormal shunting of blood through the malformation diverts blood flow away from surrounding brain tissue, potentially causing neurological deficits. The hemodynamic alterations associated with AVMs can also lead to changes in local oxygen saturation and cerebral autoregulation, impacting the overall perfusion dynamics of the brain [6]. Understanding the pathophysiology of cerebral AVMs is crucial for guiding treatment strategies and predicting clinical outcomes. Current research focuses on elucidating the mechanisms underlying AVM formation, progression, and rupture, aiming to develop targeted therapies to manage this complex vascular condition effectively.

The imaging algorithm for the work-up and follow-up of AVMs may depend on the management strategy chosen. It is important to consider that many patients diagnosed with AVMs are young and may require surveillance for several years. Therefore, if possible, magnetic resonance imaging (MRI) should be preferred to compute tomography (CT) or digital subtraction angiography (DSA) to minimize ionizing radiation exposure. Although DSA often remains central for AVM diagnosis, advances in noninvasive techniques open up multiple scenarios for the application of new diagnostic paradigms in some clinical settings. The purpose of this article is to highlight all the key features and evidence to provide new insights for diagnosing and following AVMs. In this review, we also illustrate the role of arterial spin labeling (ASL) MRI for the work-up and evaluation of AVMs both in adult and pediatric settings.

## 2. Imaging of AVMs

Imaging AVMs serves four main purposes: accurate diagnosis, prompt identification of immediate complications, risk stratification for future issues, and guidance for selecting the most effective and least risky treatment modality. Longitudinal monitoring over time aids in assessing treatment response or ensuring stability during conservative management [7].

Classifications (Table 1) are helpful in clinical practice to grade the severity and the prognosis of the disease so that we can choose the most appropriate diagnostic therapeutic and follow-up approach. First, the Spetzler–Martin classification system, introduced almost 40 years ago, categorizes AVMs on a 5-point scale based on nidus size, location in eloquent or non-eloquent brain regions, and venous drainage pattern [8]. Lawton introduced an additional classification of AVMs that integrates patient age, hemorrhagic presentation, nidal diffusivity, and deep perforating artery supply with the Spetzler–Martin classification system, which has been shown to be a better predictor of neurological outcomes after AVM surgery [9]. It aids in predicting the risk of neurologic deficits post-surgery. The simplified Spetzler–Ponce model divides AVMs into three tiers, recommending surgical resection for class A lesions, multimodality treatment for class B lesions, and observation for class C lesions. While useful for surgical risk estimation, these schemes provide limited insight into the natural history of AVMs or adverse events following alternative treatments [10]. Various classification schemes tailored to different treatment modalities have been developed. Radiosurgical models, like the Pittsburgh modified Radiosurgery-Based AVM Score (RBAS) and Virginia Radiosurgery AVM Scale, assess overall outcomes, incorporating factors such as AVM nidus volume, location, clinical history, and age. Endovascular gradings, like the Toronto, Puerto Rico, and Buffalo score and the Embocure score, focus on AVM angioarchitecture, including arterial pedicle and draining vein characteristics [11]. These schemes enhance understanding and risk assessment in the context of specific treatment approaches.

The diagnostic imaging pathway to detect AVMs is tailored to the patient’s clinical presentation. The initial diagnosis typically relies on CT or MR imaging. Non-contrast CT, while limited in assessing AVM architecture, is crucial for promptly excluding acute hemorrhage. AVM-related hemorrhage can manifest in various patterns, from intraparenchymal to subarachnoid, and is generally detectable on non-contrast CT. Even without hemorrhage, serpentine or mildly hyperattenuating structures may sometimes suggest the presence of AVMs. CT angiography (CTA) offers widespread availability and immediate assessment of post-hemorrhage. It reveals the dysplastic blood vessels comprising the AVM nidus and is particularly effective in visualizing aneurysms and stenoses [12]. However, conventional CTA’s temporal limitations hinder the resolution of AVM fistulization extent. Advanced techniques, like CT perfusion and whole-brain 4-dimensional CTA, aim to evaluate AVM hemodynamics but are not yet widely used. CT perfusion reveals distinctive patterns in peri-nidal brain tissue abnormalities, providing insights into functional arterial and venous phenomena [13]. A multicenter retrospective study focused on developing a machine learning model indicated that radiomic characteristics are a valuable addition to traditional risk factors for predicting brain AVM (bAVM) rupture studied by the segmentation of CTA images [14].

MRI, the preferred modality for assessing AVM effects on surrounding brain parenchyma, involves a comprehensive protocol. It includes T2-weighted images for nidus size and location, fluid-attenuated inversion recovery (FLAIR) sequences for peri-nidal edema, susceptibility-weighted imaging (SWI) or gradient echo sequences (GRE) for evidence of prior hemorrhage, diffusion-weighted magnetic resonance imaging (DWI) for acute ischemia, and T1-weighted images for overall anatomy and blood products [15,16]. Vessel wall imaging aids in localizing bleeding sites, aiding urgent treatment decisions. Magnetic resonance angiography (MRA), akin to CTA, helps identify AVM presence, and dynamic techniques, like time-resolved imaging of contrast kinetics (TRICKS) or time-resolved angiography with stochastic trajectories (TWISTs), provide excellent spatial and temporal resolution. MR angiography’s lack of radiation exposure is advantageous, but its sensitivity and specificity for AVM recurrence, especially in children, may be suboptimal [17].

DSA remains the gold standard for AVM diagnosis, evaluation, and surveillance, offering superior spatial and temporal resolution. Peri-nidal angiogenesis may necessitate a comprehensive six-vessel DSA for initial evaluation. Three-dimensional and multiplanar reconstructions enhance the identification of peri-nidal aneurysms and stenoses [18]. Additional imaging modalities are represented by brain perfusion scintigraphy with 123I-IMP or with 99mTc-ECD (dynamic phase and SPECT) in AVM patients, which are useful for estimating the diameter of the AVM nest as areas of increased tracer activity. SPECT images generally show the AVM nidus as a region of lower tracer accumulation, with hypoperfusion in the brain tissue adjacent to the nidus [19].

## 3. Perfusion-Weighted Imaging (PWI)

PWI MRI refers to a set of noninvasive imaging techniques designed to assess hemodynamic parameters within the brain. The primary goal of PWI is to provide real-time insights into brain function, tissue viability, angiogenesis in oncology, or vascular malformation assessment. It constitutes a valuable tool for a wide range of diseases and research. PWI encompasses multiple imaging techniques, including Dynamic Susceptibility Contrast (DSC), Dynamic Contrast-Enhanced (DCE), and arterial spin labeling (ASL). An overview of these techniques will be discussed below.

### 3.1. Dynamic Susceptibility Contrast (DSC) PWI

DSC PWI uses an exogenous contrast agent, typically an intravenous gadolinium injection. While passing through the capillary beds, this tracer will induce spin dephasing, leading to a transient drop in T2 or T2* signal intensity, correlating with tissue perfusion. DSC-PWI enables the estimation of microvasculature parameters, such as mean transit time (MTT), time to peak (TTP), cerebral blood flow (CBF), and cerebral blood volume (CBV). These parameters provide important information for the patient’s workup. For example, CBV informs microvessel density (MVD), i.e., the number of capillaries or small vessels in an area, and Microvessel Area (MVA), i.e., the total area occupied by small vessels in a defined region of interest [20].

DSC PWI has a wide range of applications. An elevated CBV in a given area compared to the controlateral side, called the “relative CBV (rCBV)”, often suggests neovascularization, helping with preoperative tumor grading [21,22]. This is a useful method for differentiating low-grade gliomas (LGGs) from high-grade gliomas (HGGs). Law et al. proposed a cutoff value of 1.75 for the rCBV ratio to differentiate LGGs from HGGs [23,24].

MVA has also been correlated with glioblastoma prognosis by identifying a predominance of glomeruloid vascular structures over capillary-type vessels [20]. In meningiomas, there is a correlation between CBV and histologic measures, both MVA and MVD. There is also a correlation between rCBV and the expression of VEGF and the Ki-67 proliferative index [25]. DSC PWI also facilitates differentiating infarcted brain tissue from salvageable tissue in ischemic stroke, aiding patient selection for therapy [26].

### 3.2. Dynamic Contrast-Enhanced (DCE) PWI

DCE PWI mainly focuses on assessing vessel permeability. Like DSC, it relies on the administration of an intravenous exogenous contrast agent, such as gadolinium [20]. First, DCE acquires pre-contrast T1 values followed by contrast-enhanced arterial input measurements, employing a T1 relaxivity approach. From these raw data, a pharmacokinetic model monitors the exchange of gadolinium between intravascular and extravascular compartments [27]. Through this process, it can calculate quantitative parameters, such as the volumetric transfer constant (K-trans), fractional plasma volume (Vp), and fractional volume of the extra-cellular extravascular space (Ve). These data help distinguish between immature blood vessels, which have high permeability, and mature blood vessels.

DCE PWI is especially useful for assessing tumor perfusion, microvascular vessel wall permeability, and extravascular–extracellular volume fraction. These parameters serve as biomarkers for tumor progression, guiding treatment planning and evaluating therapeutic response [28].

To date, several studies have used DCE MRI to detect blood–brain barrier (BBB) disruption in various neurological disorders, including stroke, brain tumor, dementia, mild cognitive impairment, and traumatic brain injury [29,30]. For assessing blood flow in AVM nidus and veins, DCE PWI seems inferior to ASL PWI [31].

### 3.3. Arterial Spin Labeling (ASL) PWI

ASL PWI provides a noninvasive measurement of CBF. This technique was introduced by Williams et al. in 1991 [32]. Unlike DSC and DCE, this technique uses labeled water from blood vessels as an endogenous tracer, eliminating the need for an intravenous contrast agent [23]. This technique uses inversion radiofrequency pulses to label water protons from blood vessels before they enter the tissue of interest. This permits the correlation of perfusion territories with collateral arteries and the quantification of each collateral artery’s contribution to brain perfusion [20]. ASL also facilitates the assessment of vascular reactivity and cerebrovascular reserve capacity within the same scan session, making it ideal for evaluating vascular disorders.

The inherent technical features of the method and what has been said so far make it clear that PWI ASL helps to assess the perfusion territories of individual cerebral arteries in conditions, such as AVMs, but also epilepsy, tumors, and neurodegenerative disorders. However, we must emphasize that it finds a central and main role in the diagnosis of ischemic cerebrovascular conditions, i.e., stroke, in which it plays a reliable and accurate role in the study of penumbra [33]. In addition to these “traditional” applications, PWI ASL is also valuable in new contexts, such as in predicting tumor vascular normalization following antiangiogenic therapy [34]. Unlike other perfusion imaging methods, ASL’s noninvasive nature makes it suitable for initial or follow-up studies, even in patient populations such as children or those with contrast allergies or renal failure.

Before turning to the dissertation on the clinical use of ASL sequences in the context of AVMs, let us briefly introduce how ASL sequences currently work [35]. In the field of ASL, the single-post labeling technique involves the application of a single radiofrequency pulse to label arterial blood water. Although conceptually simple, this approach can present problems related to the signal-to-noise ratio (SNR) and spatial coverage.

One methodological advance is undoubtedly multi-post labeling ASL, particularly 3D pseudo-continuous ASL (PCASL), which involves the application of multiple labeling pulses. This method aims to improve SNR and spatial coverage compared with single-post labeling. In addition, 3D PCASL sequences allow three-dimensional coverage, making them suitable for whole-brain perfusion imaging. In addition, ASL extends its capabilities to the realm of four-dimensional magnetic resonance angiography (MRA). By introducing a temporal dimension to the imaging process, ASL-based 4D MRA enables dynamic visualization of blood flow. This proves invaluable for the identification of vascular abnormalities and diseases, providing a comprehensive understanding of cerebral vascular dynamics over time. Overall, as stated in the guidelines by Lindner et al. published in 2023, for initial assessment, single-PLD ASL is sufficient, whereas for shunt flow characterization, multi-PLD imaging provides insights, and relative CBF allows for detection and diagnosis, while evaluation of the steal phenomenon is preferably performed using quantitative CBF data [35].

## 4. The Role of ASL MRI in Adult AVMs

### 4.1. Work-Up of AVMs

Concerning the natural history of AVMs, the overall annual rates of epilepsy and hemorrhage are 1% and 3%, respectively [36]. It is clear from this framing that the most frequent setting, especially in pediatric age, which makes us diagnose AVMs, remains the radiological work-up of acute conditions resulting from their rupture or the parenchymal irritation, leading to epilepsy [37]. The hypervascular nature of AVMs is effectively highlighted by ASL. The technique is able to reveal the increased vascularity associated with AVM lesions, showing elevated macrovascular signals both outside the arterial tree and within the nidus and venous system. ASL reveals the abnormal perfusion patterns associated with AVMs, a consequence of the direct arteriovenous shunt altering typical hemodynamics. These distinctive patterns stand out against the background of the surrounding normal brain tissue. In addition to identifying AVMs, ASL plays a crucial role in delineating vascular architecture. It helps identify the feeding arteries that supply blood to the AVM and the drainage veins responsible for transporting blood from the malformation. This information is essential for surgical planning and treatment strategies. Examination of the ASL extends to perilesional changes in the surrounding tissue, providing information on the impact of the AVM on the adjacent normal brain parenchyma. Quantitatively, ASL allows precise measurement of CBF in the context of AVMs. These quantitative data on increased blood flow in and around the malformation are critical for assessing severity and guiding therapeutic decisions [35]. Identification of venous-dominant AVMs from DVAs on MRI is difficult because of visual similarities [38,39,40,41,42,43,44], so much so that it has been suggested that developmental venous anomalies (DVAs) with shunting are actually a subgroup of AVMs. Another challenging lesion is micro-AVMs [18,45,46,47,48,49,50], with a nidus of 1 cm or smaller, which pose a diagnostic challenge, especially in acute rupture. ASL sequences in MRI, with high sensitivity, help diagnose micro-AVMs. Regarding the extent of arteriovenous shunt [51,52,53,54,55], the quantitative correlation of AVM signal intensity by ASL is a noninvasive tool. There are positive correlations between venous transit time and AVM size. High-flow arteriovenous shunts are related to increased hemorrhagic events. MRA with ASL helps study high-flow shunts, offering valuable indicators for hemorrhage prediction. The choice of treatment for AVMs depends on several factors [8,56]; stereotactic radiosurgery (SRS) planning for AVMs considers factors such as nidus size, arterial supply, and venous drainage [57]. While DSA, the gold standard, has limitations in SRS planning, noninvasive alternatives, such as ASL-based 4D MRA, provide spatiotemporal angiographic information for accurate nidus delineation in SRS planning [58,59,60].

#### 4.1.1. Differential Diagnosis of Venous-Predominant AVMs and Developmental Venous Anomalies (DVAs)

Venous-predominant AVMs are almost identical in appearance to developmental venous anomalies (DVAs) on conventional MRI imaging. In venous-predominant AVMs, the venous drainage component is particularly prominent compared to the arterial supply [38]. The epidemiology of venous-predominant AVMs is not as extensively documented as AVMs in general, but they are considered a rare subtype [39]. DVA is rather an extreme developmental anatomical variation of veins than true malformation and is composed of an irregular fan-like arrangement of small veins that converge into a larger central vein [40].

Nevertheless, there is a rare subgroup of patients in whom DVAs demonstrate shunting (transient DVAs) or in whom DVAs not only drain normal brain parenchyma but also serve as the dominant venous drainage for an AVM. In the latter case, in particular, the resulting venous overload generates symptomatology that is difficult to distinguish from that of a venous predominant AVM, such as epilepsy, headache, and vertigo [41]. Im et al. based on clinical, angiographic, surgical, and histological findings, proposed as early as 2008 to classify atypical DVAs, which had shunting as a subtype of AVM rather than as a variant of DVA or as a combined vascular malformation [42]. This paradigm remains valid today and suggests that these conditions represent discrete phenotypes in a continuous spectrum of nosologic entities that share the same pathophysiology.

Therefore, to distinguish them, a comprehensive radiological study is required. MRI is particularly useful in this regard, with ASL sequences serving as a valuable aid. In a recent study conducted by Yoo and colleagues, fufteen lesions (in thirteen patients) were divided into three groups: typical venous-dominant AVMs (time step < 2 s), an intermediate group (time step between 2.5 and 5 s), and classic developmental venous anomalies (time step > 10 s). The typical venous-dominant AVMs group showed a significantly increased ASL signal, while the group with classic DVAs did not exhibit any discernible signal. However, in the intermediate group, three out of six lesions showed a slight increase in the ASL signal, posing a further challenge [43]. Clinically, it should be mentioned that patients with coexisting AVMs and DVAs tend to have a hemorrhagic presentation. Surgically speaking, contrary to the traditional management of AVMs, in these cases it is important to preserve the draining vein through the DVA to ensure safe and sustained circulatory outflow of the brain parenchyma to avoid venous infarction [44].

#### 4.1.2. Detection of Ruptured Angiogram-Negative Micro-AVMs

Micro-AVMs are defined as a subset of pial AVMs characterized by a nidus of 1 cm or smaller [45].

Although DSA is the gold standard for the definitive diagnosis of AVMs, the diagnosis of micro-AVMs remains difficult [46]. In the acute phase of rupture due to the small size of the nidus, slow flow within the nidus, the presence of intralesional thrombosis, post-hemorrhagic vascular spasm, or the compression of the nidus by the hematoma, standard MRI may be negative. However, the dedicated ASL sequence may show high signals on the inner wall of the hematoma, which is suggestive of micro-AVMs [47].

The venous signal on ASL has been reportedly useful for diagnosing micro-AVMs, with a diagnostic sensitivity as high as 78% [48]. In this regard, it has been suggested that repeat ASL should be a useful screening tool to determine the timing of DSA due to its less invasive nature, fair sensitivity, and early onset of positivity [49]. More broadly, MRA has demonstrated its ability to produce high-quality diagnostic images for the angioarchitecture of AVMs. However, not many studies with large sample sizes have been conducted for longer periods [18].

Moreover, Yamaguchi et al. explore the relationship between the signal intensity of draining veins on SWI and venous flow, as well as the presence of intracerebral hemorrhage (ICH). Their analysis, involving 10 patients with untreated arteriovenous shunts, indicates that draining veins with hypointensity on SWI may still have normal venous flow, suggesting a potentially lower risk of ICH in the surrounding areas due to reduced venous hypertension [50].

#### 4.1.3. Assessment of the Extent of Arteriovenous Shunting

Quantitatively correlating the signal intensity of AVMs at ASL with the degree of arteriovenous shunting can be a helpful and noninvasive tool for planning the treatment approach and making accurate predictive and prognostic judgments. To investigate this, a study compared the data obtained from ASL with DSA in 40 patients. The study found that the signal intensity of the AVM showed a positive correlation with the time difference between normal and AVM venous transit times (r = 0.638, *p* < 0.001). Additionally, the signal intensity of the AVM demonstrated a positive correlation with the size of the AVM (r = 0.561, *p* < 0.001) [51]. AVMs with high-flow arteriovenous shunts are associated with increased perioperative hemorrhagic events and a higher risk of incomplete obliteration [52,53,54]. MRA based on ASL can be used to study the high-flow arteriovenous shunt in AVMs. A retrospective study correlated the signal intensity (SI) ratio of the draining vein achieved by this procedure to the arteriovenous transit time measured by DSA. It was found that this approach can be used as a useful indicator of the high-flow shunt of AVMs and that draining veins with the highest SI ratio were associated with the occurrence of hemorrhages [55].

#### 4.1.4. Integration of ASL into Stereotactic Radiosurgery (SRS) Planning for AVMs

The choice of treatment for AVMs depends on several factors, including the location/size of the AVM nidus, arterial supply and venous drainage, and symptomatology [8]. Stereotactic radiosurgery (SRS) has been shown to be an effective treatment technique for AVMs [56]. The procedure involves the application of a stereotactic frame to the patient’s skull and the following acquisition of imaging studies. The results of these studies are then transferred to the planning software. The imaging aims to establish the target volume, which is crucial for subsequent planning. It has been observed that the delineation of the target volume remains a significant potential source of error in SRS treatment [57]. Imaging AVMs can be performed using various techniques. As mentioned above, for dynamic visualization of cerebral vasculature, DSA is considered the gold standard. However, it provides only lateral and anteroposterior projections of the nidus for SRS planning. This limitation is overcome by techniques such as 4D contrast-enhanced MR angiography (4D CE-MRA), a time-resolved technique that provides dynamic 3D angiographic data [58]. Specifically, 4D ASL-based MRA is a noninvasive technique known for its capability to produce high temporal resolution angiographic data [59]. A study evaluated the combination of 4D ASL and 4D CE-MRA as a prospective alternative to DSA to delineate the nidus of an AVM in planning SRS. Indeed, the combined use of the two techniques provided sufficient spatiotemporal angiographic information to delineate the nests of AVMs [60].

### 4.2. Post-Treatment Follow-Up of AVMs

It is crucial to follow up with patients after the treatment because not all malformations obliterate at the same time and with the same effectiveness. One study shows that early obliteration, defined as ≤18 months after SRS, was more common in patients whose AVMs were smaller and located in the frontal lobe, basal ganglia, or cerebellum, had deep venous drainage, and had received a marginal dose > 24 Gy [61]. The post-treatment follow-up of arteriovenous malformations (AVMs) involves a comprehensive approach, incorporating various imaging modalities to assess treatment outcomes and detect potential complications. Evaluating the efficacy of embolization, microsurgical resection, or stereotactic radiosurgery (SRS) requires an accurate measurement of shunt reduction and the detection of residual AVMs.

MRI with pulsed ASL (PASL) emerges as a promising noninvasive tool for assessing post-embolization shunt reduction, showing reliable results and fair agreement with digital subtraction angiography (DSA) [62,63]. Microsurgical resection proves effective, emphasizing the superiority of well-selected cases in minimizing long-term morbidity [64,65]. In the context of stereotactic radiosurgery (SRS), the focus shifts to detecting residual AVMs over 2 to 5 years [66], with arterial spin labeling in MRI demonstrating high sensitivity and specificity. This approach reduces the need for frequent invasive angiographic procedures during follow-up. Furthermore, the significance of peri-nidal T2 hyperintensity in predicting AVM obliteration after SRS adds another layer to post-treatment assessment [67,68]. Collectively, these findings underscore the importance of a combined imaging approach, incorporating PASL and ASL in MRI, for a comprehensive and effective post-treatment follow-up in AVM management [69,70].

#### 4.2.1. Assessment of the Extent of Arteriovenous Shunting following Embolization

A study using MRI with pulsed arterial spin labeling (PASL) measured the relative signal of the post-embolization shunt over 1.75 s and six slices covering the lesion. PASL results demonstrate a fair agreement with DSA judgments. In the case of total or subtotal shunt occlusion, PASL indicates a reduction between 69% and 92%, while in the case of minimal shunt reduction, it ranges from −6% to 35%. The PASL method proves to be quite reproducible, with a maximum deviation of 2%, offering a viable alternative to conventional angiography [62]. Alaraj et al. also applied PASL to evaluate post-embolization shunt reduction [63]. By determining the relative shunt signal before and after embolization, the study established a fair agreement between PASL and DSA. PASL demonstrated significant shunt reduction in cases of total or subtotal occlusion, providing reliable measurements (with a maximum deviation of 2%) and demonstrating its effectiveness in evaluating shunt reduction after embolization of AVMs and dural arteriovenous fistulas (DAVFs).

#### 4.2.2. Assessment of the Extent of Arteriovenous Shunting following Surgery

The assessment of the extent of arteriovenous shunting following surgery is a critical aspect of postoperative care and monitoring. There are no clear studies in the literature analyzing this aspect; more specifically, we can state that the tendency to evaluate postoperative shunting is to use angiography [64].

In any case, an extremely interesting study of 288 patients demonstrated the superiority of well-selected microsurgical cases over multimodal interventions or conservative treatment alone, as observed in the famous and criticized ARUBA study [65].

#### 4.2.3. Detection of Residual AVMs following SRS

Contrasting with the instant exclusion of AVMs following embolization or surgery, SRS typically induces AVM obliteration within 2 to 5 years post-procedure [66]. Consequently, the follow-up protocols diverge from AVMs treated via surgery or embolization to confirm treatment efficacy or the necessity for further intervention. In this context, the need for recurrent radiological follow-ups over several years advocates for noninvasive techniques. AVM volume and radiation dose are the most significant factors that impact the obliteration after SRS [67]. In a retrospective series published by Kodera et al., including seven patients, ASL demonstrated a correlation with DSA in detecting residual persistent AVM shunts [68]. Similarly, Heit et al. conducted a retrospective cohort study involving 15 patients, employing both ASL and DSA for follow-up [69]. Their findings echoed those of Kodera’s study, confirming ASL’s high sensitivity and specificity in diagnosing residual shunts. Both research teams concluded that ASL could serve as a valuable tool in determining the optimal timing for a single DSA during follow-up. They emphasized that while ASL does not replace DSA, it reduces the necessity for multiple DSAs throughout the follow-up period.

In this regard, it is pertinent to recall the significance of peri-nidal T2 hyperintensity in predicting the obliteration of AVMs after SRS. A study conducted on 62 patients demonstrated that this indicator holds statistical significance worth *p* = 0.007, with a sensitivity of 66.7% and an accuracy of 60%. However, it should be noted that the specificity of this indicator is relatively low at 20%. Nonetheless, it may be employed as a prognostic sign of complete obliteration of the AVM nidus after SRS [70].

## 5. The Role of ASL MRI in Pediatric AVMs

AVMs in the pediatric population (pAVMs) account for 3% of all AVMs. Nevertheless, pAVMs are responsible for 80% of non-traumatic brain hematomas [71,72]. Furthermore, pAVMs tend to rupture more frequently than AVMs in the adult population, and they are more likely to reappear after DSA-proved complete obliteration, reinforcing the need for prolonged follow-up and the drawbacks of invasive imaging in the setting of a healed shunt [73]. In this population with high life expectancy and higher risks of rupture and recurrence, ASL seems to be an appealing alternative to CTA, single-photon emission computed tomography (SPECT), CE-MRA, and DSA, considering the long-term risks of ionizing radiation, injection of an exogenous contrast agents, multiple exposures to general anesthesia, and neurologic adverse events.

In a prospective cohort study of 120 children presenting with intracerebral hemorrhage, Hak et al. showed that ASL is effective at diagnosing arteriovenous shunts, such as AVMs and DAVFs [74]. Indeed, they report a sensitivity of 0.90 and a specificity of 0.97. Hence, they propose initiating patient workup with a non-contrast study including at least time-of-flight (TOF) MRA and ASL sequences. In case of positive ASL, they suggest proceeding with post-contrast imaging, including 4D CE-MRA and gadolinium-enhanced 3D T1-weighted MRI, for anatomic evaluation of the AVM. This scheme will not spare DSA but will eventually reduce the need for contrast in some patients. In a second study, Hak et al. [72] investigated in a prospective cohort of 59 patients the role of ASL for the longitudinal follow-up before treatment and after complete obliteration of pAVMs by analyzing CBF variations regardless of the treatment option, such as unimodal or multimodal surgery, embolization, and/or SRS. They point out that CBF measured in ASL is a reliable biomarker to detect AVM recurrence. Furthermore, they showed that a decrease in CBF of 50 mL/100 mg/min seen in ASL at one year after SRS is associated with a three-fold increase in the rate of eventual obliteration after three years of treatment, thus serving as a prognostic index.

## 6. Future Perspectives

### 6.1. Non-Contrast-Enhanced (NCE) Time-Resolved 4D Dynamic MRA at 7T

Ultrahigh fields offer an increased resolution for neurovascular imaging, providing high spatial and temporal resolutions, a detailed representation of vascular architecture, and dynamic blood flow patterns [75]. Non-contrast-enhanced 4D MRA (NCE 4D MRA) based on ASL shows potential in characterizing cerebrovascular hemodynamics in cerebrovascular disorders. A prospective study involving six healthy volunteers and eight patients with AVMs analyzed the feasibility of NCE 4D MRA using a 3D Cartesian trajectory and a 7T stack-of-stars (SOS) radial golden angle trajectory, demonstrating excellent delineation of AVM features, especially the draining veins [76]. Togao et al. have also evaluated the usefulness of 4D MRA based on pseudo-continuous super-selective ASL combined with keyhole and view sharing (4D-S-PACK) in assessing the hemodynamics of cerebral AVMs. They concluded that the method provides perfect identification of the feeding arteries of cerebral AVMs [77]. In light of this evidence, new studies on this noninvasive clinical tool for assessing the hemodynamics of cerebral AVMs represent an enlightening research direction and should be implemented.

### 6.2. Toward Noninvasive Diagnosis and Follow-Up of pAVMs

Advances in medical imaging techniques have paved the way for noninvasive diagnosis and follow-up of AVMs for several years [78]. This noninvasive diagnostic approach is most important in the pediatric population, where minimizing procedural risks is crucial [79]. Furthermore, these noninvasive imaging modalities facilitate longitudinal monitoring, allowing efficient follow-up assessments of AVM morphology and hemodynamics. Indeed, a retrospective study to evaluate the relevance of ALS sequences for diagnosis and follow-up of pAVMs studied 21 pediatric patients treated with SRS or embolization and undergoing DSA and pseudo-continuous ASL MRI. ASL MRI effectively revealed the location of the nidus and its patency after treatment [71]. Like many of the brain’s vascular lesions, general risk factor evaluation does not allow us to make a reliable prognostic–predictive judgment ab initio. This is where machine learning and artificial intelligence (AI) tools come to our aid, which, starting with imaging, may help stratify the risk of bleeding in individual patients, helping the physician to choose the correct treatment approach [80].

The shift toward noninvasive diagnosis and follow-up represents a significant stride in pediatric neuroimaging, offering a safer and more patient-friendly means of managing brain arteriovenous malformations in the younger demographic.

## 7. Limitations

*Comparison of ASL with DSA*

ASL stands out as a promising noninvasive tool for pediatric and adult AVM evaluation, yet a nuanced understanding of its advantages and limitations is crucial. Although ASL’s endogenous tracer and safety profile make it an appealing choice, it is important to note potential challenges (Table 2).

ASL’s reliability in assessing venous drainage might be compromised due to spin inversion recovery, leading to signal reduction [81].

Interstudy variation in MRI parameters and uncertain sensitivity for specific AVM features introduce complexities in its application [31]. Detecting AVMs in challenging locations, such as the apical cranial convexity, becomes more arduous [82] because there is a risk of underestimating total blood flow [62], demanding careful consideration.

A systematic review by Ramachandran and colleagues, which examined 289 patients with intracranial AVMs, compared ASL performance with the current gold standard of DSA. ASL demonstrated a sensitivity ranging from 84.6 to 100% and a specificity ranging from 93.3 to 100% in identifying feeder arteries, and it also a strong performance in detecting arteriovenous shunting with a sensitivity ranging from 91.7 to 100% and a specificity ranging from 90 to 100%. ASL can identify the location and size of nests, like DSA. However, other studies have shown relatively inferior performance in delineating venous drainage [81,83]. Finally, two of the papers included in this review demonstrated 100% sensitivity of ASL in identifying residual or obliterated AVMs after SRS [84].

In navigating these challenges, ASL offers a noninvasive avenue with undeniable merits, such as functional assessment and arterial feeder identification [85].

Acknowledging its limitations ensures a balanced approach to harnessing ASL’s potential for AVM evaluation. Nonetheless, larger-scale prospective studies need to be conducted to evaluate the diagnostic performance of ASL.

## 8. Conclusions

ASL emerges as a promising noninvasive tool for both pediatric and adult AVM evaluation, offering advantages such as functional assessment and arterial feeder identification. The natural history of AVMs underscores the importance of their diagnosis in the context of acute conditions resulting from rupture or parenchymal irritation leading to epilepsy, especially in pediatric cases. While general risk factor evaluation does not provide a reliable prognostic judgment, advancements in statistical, machine learning, and artificial intelligence tools, particularly those applied to imaging, offer the potential to stratify bleeding risk in individual patients. Distinguishing venous-predominant AVMs from developmental venous anomalies (DVAs) poses a challenge due to their similar appearance on conventional MRI imaging. Comprehensive radiological studies, especially utilizing MRI with arterial spin labeling (ASL) sequences, prove valuable in this distinction. The rare subgroup of DVAs with shunting or serving as dominant venous drainage for an AVM requires careful consideration and imaging evaluation. Micro-AVMs also present diagnostic challenges, particularly in the acute phase of rupture. ASL sequences, specifically dedicated ASL, enhance the detection of ruptured micro-AVMs by revealing high signals on the inner wall of hematomas. Repeat ASL serves as a useful screening tool for determining the timing of digital subtraction angiography (DSA).

The integration of ASL into stereotactic radiosurgery (SRS) planning for AVMs provides valuable spatiotemporal angiographic information, aiding in the delineation of AVM nests. Post-treatment follow-up, crucial due to variable obliteration times, involves assessing the extent of arteriovenous shunting, detecting residual AVMs, and utilizing peri-nidal T2 hyperintensity as a prognostic indicator for complete obliteration. However, a nuanced understanding of its limitations is crucial. Challenges include potential signal reduction in assessing venous drainage, interstudy variation in MRI parameters, and uncertainty in sensitivity for specific AVM features. Overall, advancements in imaging techniques, especially those incorporating ASL, offer enhanced diagnostic capabilities, aiding in the accurate diagnosis, classification, and management of cerebral arteriovenous malformations. Ongoing research and technological innovations continue to improve our understanding and clinical approach to these complex vascular lesions.

## Figures and Tables

**Table 1:** Classifications of AVMs and purposes.

Classification	Purpose/Modality	Key Factors Assessed
Spetzler–Martin Grading System	Surgical risk estimation	Nidus size, location in brain, and venous drainage
Spetzler–Ponce Model	Surgical guidance	Categorizes AVMs into three tiers based on Spetzler–Martin grades
Supplementary Grading Scale by Lawton and Young	Predict neurological outcome and refine patient selection	Patient age, hemorrhagic presentation, nidal diffuseness, and deep perforating artery supply
Pittsburgh Modified RBAS	Radiosurgery outcomes	Nidus volume, location, and clinical factors (history, age)
Virginia Radiosurgery AVM Scale	Radiosurgery outcomes	Nidus volume, location, and clinical factors (history)
Toronto, Puerto Rico, and Buffalo Score	Endovascular assessment	AVM angioarchitecture, arterial pedicles, and draining veins
Embocure Scor	Endovascular assessment	AVM angioarchitecture, arterial pedicles, and draining veins

**Table 2:** ASL vs. DSA comparison; assessment of ASL limitation.

Characteristics Analyzed	Advantages of ASL	Advantages of DSA	Disadvantages of ASL	Disadvantages of DSA
Endogenous Tracer Usage (water)	++	−	−	Contrast agent required; risk of complications
Safety as a Noninvasive Alternative	+	−	−	Invasive procedure; potential for vascular complications
Functional AVM Assessment	Provides functional assessment	−	Reduced reliability for venous drainage assessment (spin inversion)	Limited functional information; anatomical focus
Identification of Arterial Feeders	−/+	−	Difficulty in detecting AVMs in specific locations	Excellent identification of arterial feeders; comprehensive view
Characterization of Low-Flow Segments	−	−	Underestimation of total blood flow in AVMs	May not provide detailed information on low-flow segments
Mitigation of Overestimation Risk	−/+	−	Limitations in the immediate postintervention period	Potential for overestimation due to strong contrast agent injection
Identification Despite Mass Effect	−	−	Challenges in interpretation due to inhomogeneities	Excellent for overcoming mass effect; direct visualization

+ (moderate advantages), ++ (strong advantages), −/+ (indifferente) and – (not reported).
